# Occult contralateral inguinal hernias: what is their true incidence and should they be repaired?

**DOI:** 10.1007/s00464-018-6528-y

**Published:** 2018-10-16

**Authors:** Joey A. Jarrard, Michael R. Arroyo, B. Todd Moore

**Affiliations:** 1 Department of Surgery, Saint Luke’s Hospital of Kansas City, Kansas City, MO USA; 2 Saint Luke’s Hospital of Kansas City, 4320 Wornall Rd, Medical Plaza 1, Ste 530, Kansas City, MO 64111 USA

**Keywords:** Inguinal hernia, Occult hernia, Robotic hernia repair, Transabdominal preperitoneal repair

## Abstract

**Background:**

The true incidence of occult contralateral inguinal hernia is unknown; however, when found, there exists controversy as to whether or not they should be repaired. The aim of our study is to identify the incidence of contralateral incidental inguinal hernias in our surgical population, compare our results to previous studies timelining occult hernia identification to repair need, and generate debate as to whether incidental contralateral hernias should be repaired at the index operation.

**Methods:**

We reviewed the charts of 297 consecutive patients undergoing robotic inguinal hernia repair between October 2014 and April 2018 at a single facility. By comparing preoperative physical examination to intraoperative findings, we determined the number of occult contralateral inguinal hernias in our patient population.

**Results:**

Of 297 patients, 158 (53.2%) presented with a right inguinal hernia, 90 (30.3%) presented with a left inguinal hernia, and 49 (16.5%) presented with bilateral inguinal hernias. Forty-seven of the 297 patients (15.8%) were found to have an incidental contralateral inguinal hernia. Excluding patients with known bilateral inguinal hernias, 20% of patients with a left inguinal hernia were found to have an occult right inguinal hernia and 18.4% of patients with a right inguinal hernia were found to have an occult left inguinal hernia.

**Conclusions:**

The true incidence of occult contralateral inguinal hernia may be higher than originally thought. When inguinal hernia repair is performed through a transabdominal approach, these occult hernias may be easily addressed during the same operation without additional skin incisions. This may ultimately prevent the morbidity of developing a metachronous hernia that requires repair.

Inguinal hernia repair is historically one of the most commonly performed operations by general surgeons on a global scale. Through evidence-based proposals to improve patient outcomes, we continue to see advancements in surgical technique and change in best practice recommendations. This has led to a multitude of methods, theories, and strategies all with varying recurrence and complication rates as well as presumed patient advantages in the reported literature [1]. With the advent of minimally invasive approaches for inguinal hernia repair, the identification of occult, contralateral inguinal hernias not detected on physical examination has become a common finding during repairs for symptomatic hernias. The true incidence of contralateral incidental inguinal hernias is unknown. With minimally invasive techniques, access to and repair of the occult defect can be achieved with limited adjustments to the surgical approach. This potentially obviates the patient from future symptoms, morbidity, and an additional operation. The traditional dogma of avoiding repair of asymptomatic hernias applies to open techniques and should be challenged. The aim of our study is to identify the incidence of contralateral incidental inguinal hernias in our surgical population, compare our results to previous studies timelining occult hernia identification to repair need, and generate debate as to whether incidental contralateral hernias should be repaired at the index operation.

## Materials and methods

Our facility’s Epic software was used to retrospectively review the charts of 297 consecutive patients undergoing robotic inguinal hernia repair from October 2014 to April 2018. A single surgeon performed all procedures at the same institution. The preoperative history and physical as well as the operative report were reviewed for each patient. We determined whether each patient was felt to have right, left, or bilateral inguinal hernias based on the preoperative physical examination. This information was compared to intraoperative findings in the surgeon’s operative report to determine whether an incidental inguinal hernia was present. All inguinal hernias were approached with the same technique, using three trocars on the da Vinci Si platform: a transabdominal preperitoneal (TAPP) dissection was performed and mesh was placed and secured with Vicryl suture. For patients presenting with a unilateral hernia, the contralateral side was routinely inspected for an incidental hernia and repaired if present. During this time period, there were two patients who underwent open repair due to the presence of strangulated bowel requiring resection. There were three patients who underwent laparoscopic repair due to their presenting with incarceration after hours when the robotic platform was not available. These five patients were not assessed for contralateral hernia and therefore not included in this analysis.

## Results

Our patient population had an average BMI of 26.48 and was at an average 60.2 years of age at the time of their operation. Of the 297 patients, 158 (53.2%) presented with a right inguinal hernia, 90 (30.3%) presented with a left inguinal hernia, and 49 (16.5%) presented with bilateral inguinal hernias. Forty-seven of the 297 patients (15.8%) were found to have an incidental contralateral inguinal hernia. Excluding patients with known bilateral inguinal hernias, 20% of patients with a left inguinal hernia were found to have an occult right inguinal hernia and 18.4% of patients with a right inguinal hernia were found to have an occult left inguinal hernia. Of patients found to have a unilateral right inguinal hernia, 16.3% had previously undergone repair of the left side. Of those found to have a unilateral left inguinal hernia, 29.2% had previously undergone repair of the right side. Repairing the contralateral side required no additional incisions and did not affect the decision of when to discharge the patient in any case. The duration of each procedure was not available for review in every patient and was therefore not included in our analysis. However, the primary surgeon felt that repair of an incidental hernia did not significantly increase the length of the procedure.

## Discussion

The incidence of contralateral inguinal hernias has been examined multiple times in the literature [2–4]. Some studies were structured to evaluate both groins at the initial operation, while others followed patients longitudinally for the development of metachronous contralateral hernia. Subsequently, the reported rate of contralateral hernia has ranged from 8 to 13%. In our study, we routinely examined both sides transabdominally for the presence hernias and found occult hernias at a rate slightly higher than previously reported. This review brings to light the significant rate of contralateral occult inguinal hernia and draws consideration as to whether or not these hernias should be repaired routinely (Table 1).

**Table 1:** Hernia laterality at presentation, occult hernias found, and number with prior repair

Laterality^a^	# Patients	# Occult contralateral hernia	# Unilateral with prior contralateral repair^b^
Right	158 (53.2%)	29 (18.4%)	21 (16.3%)
Left	90 (30.3%)	18 (20%)	21 (29.2%)
Bilateral	49 (16.5%)	NA	NA
Total	297	47	42 (20.9%)

*NA* not applicable

^a^Laterality identified preoperatively on examination

^b^Number of patients found to have unilateral hernia intraoperatively, but had the other side repaired previously

Using a transabdominal preperitoneal technique for inguinal hernia repair, evaluation and mesh coverage of all defects of the myopectineal orifice, including incidental contralateral hernias, can be performed without making additional skin incisions. This cannot be said for contralateral repair via an open technique. As data for minimally invasive approaches to inguinal hernia repair continue to accumulate, outcomes are expected to be at least equal to those of open repairs, given appropriate expertise and training. Studies have shown a lower incidence of chronic groin pain, infection, and other wound complications with minimally invasive approaches while maintaining a similar recurrence rate [5, 6]. Even though the minimally invasive approach carries a low morbidity rate, current literature does not support routine preperitoneal dissection to identify and repair occult hernias. However, if a hernia is identified through the transabdominal approach, the benefit of repair is felt to outweigh the risk associated with a preperitoneal dissection [7]. Certainly, a strong argument can be made against routinely making a separate groin incision to evaluate for and repair asymptomatic hernias, but given our growing experience with minimally invasive techniques, this argument should not hold true for the transabdominal preperitoneal approach. In all cases, the possibility of identifying and repairing an occult hernia should be discussed with the patient in the preoperative consent.

Morbidly obese patients present a unique problem in that physical examination may be unreliable and suspected bilateral inguinal hernias may actually represent lymphatic or adipose tissue. The TAPP approach to inguinal hernia repair offers the benefit of examining the myopectineal orifice bilaterally with high sensitivity, while potentially avoiding difficult groin incisions and subsequent wound complications that are more prevalent in the obese population [8]. Performing this procedure on the da Vinci robotic platform significantly improves surgeon dexterity and ergonomics, particularly in patients with higher BMI and thicker abdominal walls [9].

In 2012, Mizrahi and Parker published a systematic review in the Archives of Surgery regarding the management of asymptomatic inguinal hernias [10]. They concluded that elective repair of asymptomatic inguinal hernias could be done with little morbidity and that watchful waiting, while safe, resulted in a high rate of eventual repair. These conclusions were made in a population undergoing open hernia repair. An interesting finding in our review is that patients that presented with right and left unilateral inguinal hernias had prior contralateral repairs 16 and 29% of the time, respectively. Thus, in addition to the nearly 16% of patients found to have an occult contralateral hernia, a significant number of the remaining patients were presenting for their second operation for a metachronous contralateral hernia not found at the time of their initial surgery. Given the high incidence of occult hernia found in our patient population and the observed progression rate to need for repair, we argue that delaying repair, though it may be safe, will often result in a second operation in a patient that may or may not be as healthy as they were at their index procedure.

There are some weaknesses to our study. Our patient population is relatively small and may not adequately represent findings that could be extrapolated to a larger scale. The average age of our patients was over 60 years, and the average BMI was 26. These and other patient factors will certainly vary by region and practice, which may in turn affect rates of contralateral inguinal hernia. Occult hernias by definition represent hernias not identified on physical examination. Palpation of a subtle inguinal hernia can be subjective and will vary among examiners, which could explain the variation in reported rates of occult inguinal hernia. Furthermore, the transabdominal approach may not identify small hernias containing only preperitoneal fat or lipomas of the cord, making the true rate of occult hernia even higher than what was seen in our study. Despite these variations in reported rate of occult inguinal hernia, we know that the rate is significant. Given the minimal added morbidity and effort required to repair a contralateral hernia via the TAPP approach, we recommend routine repair.

## References

[1] Lau WY (2002). History of treatment of groin hernia. World J Surg.

[2] Tackett LD, Breuer CK, Luks FI, Caldamone AA, Breuer JG, DeLuca FG, Caesar RE, Efthemiou E, Wesselhoeft CW (1999). Incidence of contralateral inguinal hernia: a prospective analysis. J Pediatr Surg.

[3] Zheng R, Altieri MS, Yang J, Chen H, Pryor AD, Bates A, Talamini MA, Telem DA (2017). Long-term incidence of contralateral primary hernia repair following unilateral inguinal hernia repair in a cohort of 32,834 patients. Surg Endosc.

[4] Koehler RH (2002). Diagnosing the occult contralateral inguinal hernia. Surg Endosc.

[5] Weber-Sanchez A, Weber-Alvarez P, Garteiz-Martinez D (2016). Laparoscopy and bilateral inguinal hernias. J Surg Transpl Sci.

[6] McCormack K, Scott NW, Go PM, Ross S, Grant AM (2003). Laparoscopic techniques versus open techniques for inguinal hernia repair. Cochrane Database Syst Rev.

[7] The HerniaSurge Group (2018). International guidelines for groin hernia management. Hernia.

[8] Pierpont Y, Dinh T, Salas R, Johnson E, Wright T, Robson M, Payne W (2014). Obesity and surgical wound healing: a current review. ISRN Obes.

[9] Donkor C, Gonzalez A, Gallas M, Helbig M, Weinstein C, Rodriguez J (2017) Current perspectives in robotic hernia repair. 10.2147/RSRR.S101809 10.2147/RSRR.S101809 PMC6193421 30697564

[10] Mizrahi H, Parker M (2012). Management of asymptomatic inguinal hernia—a systematic review of the evidence. Arch Surg.

